# Combination of Posaconazole and Amphotericin B in the Treatment of *Candida glabrata* Biofilms

**DOI:** 10.3390/microorganisms6040123

**Published:** 2018-12-04

**Authors:** Célia F. Rodrigues, Diana F. Alves, Mariana Henriques

**Affiliations:** 1 Centre of Biological Engineering, Laboratório de Investigação em Biofilmes Rosário Oliveira, University of Minho, 4710-057 Braga, Portugal; dianalves@ceb.uminho.pt (D.F.A.); mcrh@deb.uminho.pt (M.H.); 2 Laboratory for Process Engineering Environment Biotechnology and Energy—Department of Chemical Engineering, Faculty of Engineering, University of Porto, 4200-465 Porto, Portugal

**Keywords:** *Candida*, posaconazole, amphotericin B, biofilms, matrix, antifungal drug, antifungal combination, resistance, *Candida glabrata*

## Abstract

Candidemia cases have been increasing, especially among immunosuppressed patients. *Candida glabrata* is one of the most resistant *Candida* species, especially to the azole drugs, resulting in a high demand for therapeutic alternatives. The minimum inhibitory concentration (MIC), minimum fungicidal concentration (MFC), and minimum biofilm eradication concentration (MBEC) were determined for posaconazole (Pcz) and amphotericin B (AmB). The drug combinations of both drugs were evaluated on pre-formed biofilms of *C. glabrata* ATCC 2001, through XTT (2,3-bis (2-methoxy-4-nitro-5-sulfophenyl)-2H-tetrazolium-5-carboxanilide) assay, colony forming units (CFU), crystal violet, and the fractional inhibitory concentration index (FICI). *C. glabrata* revealed higher susceptibility and biofilm reduction in the presence of AmB alone, but both drugs revealed a good capacity in the biomass elimination. In the majority of the tested combinations, the interactions were defined as indifferent (FICI ≤ 4). The combination of the two drugs does not seem to bring a clear advantage in the treatment of biofilms of *C. glabrata*.

## 1. Introduction

Systemic fungal infections caused by *Candida* species have increased in the last decade, in parallel with the number of immunocompromised patients [1,2]. *Candida glabrata* is currently the second most common cause of candidemia in the United States and the third in Europe [3,4]. Despite not having the capacity to form filaments or to secrete proteases, *C. glabrata* has important virulence factors, such as secretion of phospholipases, lipases, and hemolysins, and especially their ability to form biofilms [1,5]. Biofilms are surface-associated communities of microorganisms embedded in an extracellular matrix which confers protection and consequently a major resistance to antifungal therapy [6,7,8]. The formation of *Candida* species biofilms raises significant clinical issues because of an additional increase in antifungal drug resistance, as well as increased evasion of host immune defenses. Furthermore, biofilm development on medical devices can cause the failure of the device and may turn into a source of future infection [9]. 

This increase in *C. glabrata* infections is related to an inherent low susceptibility to azoles [10,11], and can also be a result of rare mutations that are selected by drug pressure [12]. Accordingly, the cases of fluconazole-refractory disease (resistance to fluconazole) associated with *C. glabrata* are common in candidiasis. Therefore, the treatment of these infections relies on other azoles (e.g., voriconazole and posaconazole-Pcz) or other classes of antifungal agents, such as polyenes (e.g., amphotericin B-AmB) and echinocandins [2,13]. However, resistance to polyenes has been reported on *C. glabrata* [14,15,16,17,18], as well as resistance or low susceptibility to Pcz [19,20,21].

Pcz is a lipophilic broad-spectrum triazole drug introduced in 2007 [22,23], and the most recent guidelines for the treatment of fungal infections indicate Pcz as a valuable choice in the therapy of *Candida* intravascular infections, oropharyngeal or esophageal candidiasis [2,24]. Therapeutically, Pcz offers advantages over fluconazole and voriconazole, since its activity seems to be less affected by either mutation in *ERG11* (ergosterol gene) or the overexpression of specific efflux pumps [25]. Overall, these data would indicate a beneficial effect of Pcz in clinical circumstances in which other triazoles failed.

AmB is a polyene produced by *Streptomyces nodosus* [26,27], characterized by a macrocyclic ring lactone, that has demonstrated to be effective for the treatment of candidemia in nonneutropenic patients and urinary tract infections, especially due to *C. glabrata* and *C. krusei* [2]. Therefore, the main aim of this research was to evaluate the *in vitro* activity of the combination of one azole (Pcz) and one polyene (AmB) against *C. glabrata* mature biofilms.

## 2. Materials and Methods 

### 2.1. Organism and Growth Conditions

The reference strain from the American Type Culture Collection, *C. glabrata* ATCC 2001, was used in this study. For each experiment, *C. glabrata* ATCC 2001 was subcultured on Sabouraud dextrose agar (SDA) (Merck, Darmstadt, Germany), for 24 h at 37 °C. Cells were then inoculated in Sabouraud dextrose broth (SDB) (Merck, Darmstadt, Germany), and incubated for 18 h at 37 °C under agitation at 120 rpm. After incubation, the cells were harvested by centrifugation at 3000 *g* for 10 min at 4 °C, and washed twice with Phosphate Buffered Saline (PBS, pH = 7.5). Pellets were then suspended in RPMI-1640 (Sigma-Aldrich, St. Louis, MI, USA), and the cellular density was adjusted to 1 × 10^5^ cells/mL, using a Neubauer counting chamber [28].

### 2.2. Antifungal Drugs

Posaconazole was kindly provided by Merck^®^, in its pure compound. Amphotericin B was purchased in Sigma^®^ (Sigma-Aldrich, purity ~80%). Aliquots of 5000 mg/L were prepared using dimethyl-sulfoxide (DMSO) for both drugs, and the final concentrations were prepared in RPMI-1640.

All susceptibility tests were performed according to the European Committee on Antimicrobial Susceptibility Testing (EUCAST) guidelines [29,30].

### 2.3. Minimum Inhibitory Concentrations (MICs) 

The tested concentrations of Pcz and AmB were prepared in RPMI-1640 (pH = 7). The inoculum was prepared by suspending five distinct colonies, ≥1 mm diameter from 24 h cultures, in at least 3 mL of sterile distilled water. The inoculum was then suspended by vigorous shaking on a vortex mixer for 15 s, the cell density was adjusted to the density of a 0.5 McFarland standard, and adding sterile distilled water as required, giving a yeast suspension of 1–5 × 10^6^ colony forming units (CFUs)/mL. A working suspension was prepared by a dilution of the standardised suspension in sterile distilled water to yield 1–5 × 10^5^ CFU/mL. The 96-well-plate was prepared with 100 μL of cell suspension and 100 μL of both antifungal agents (0.2 to 1 mg/L for Pcz, and 0.25 to 2 for AmB—2 × concentrated) and incubated at 37 °C during 18–48 h. Positive controls without Pcz and AmB were also performed. According to the EUCAST, the MIC was determined as the lowest concentration (recorded in mg/L) of the drug that inhibits the growth of the yeasts to a predefined degree: 90% in the case of AmB (polyenes) and 50% for posaconazole (azoles) [29,30]. The results were visualized by spectrometry at 530 nm [29,30].

### 2.4. Minimum Fungicidal Concentration (MFC)

In addition to the previous step, 20 µL of each cell suspension treated with Pcz and AmB was recovered to a new well, and serial decimal dilutions in PBS were plated onto SDA. Agar plates were incubated for 24 h at 37 °C and the total number of CFUs were determined. The results were presented as Log_10_ CFU per area (Log_10_ CFUs/cm^2^) [28,29,30]. 

### 2.5. Minimum Biofilm Eradication Concentration (MBEC)

Standardized cell suspensions (1 × 10^5^ cells/mL) were placed into selected wells of 96-wells polystyrene microtiter plates (200 μL) (Orange Scientific, Braine-l`Alleud, Belgium). RPMI-1640 was used without cells, but with an antifungal agent as a negative control. As positive control, cell suspensions were tested without an antifungal agent. At 24 h, 100 μL of RPMI 1640 was removed and an equal volume of fresh RPMI 1640 plus the antifungal concentration were added (Pcz: 200; 300 and 600 mg/L; AmB: 0.5; 1; 2; and 4 mg/L, 2 × concentrated). The plates were incubated at 37 °C for an additional 24 h at 120 rpm. The number of cultivable cells on biofilms was determined by the enumeration of CFUs. To achieve this, the mediums were aspirated, and biofilms were washed once with 200 μL of PBS to remove non-adherent cells. Then, biofilms were scraped from the wells and the suspensions were vigorously vortexed for 2 min to disaggregate cells from the matrix. Serial decimal dilutions in PBS were plated on SDA and incubated for 24 h at 37 °C. The results were presented as Log_10_ CFU per area (Log_10_ CFUs/cm^2^) [13,29,30].

### 2.6. XTT Reduction and Checkerboard Assay 

Biofilms were formed as explained previously. The metabolic activity of the biofilms was measured by assaying 2,3-bis (2-methoxy-4-nitro-5-sulfophenyl)-2H-tetrazolium-5-carboxanilide (XTT) reduction, a reaction catalyzed by mitochondrial dehydrogenases, as described before [31]. Briefly, biofilms were washed with sterile ultrapure water and were then incubated with 100 μg/mL XTT and 10 μg/mL PMS at 37 °C for 3 h at 120 rpm. Following this, the optical density (OD) was measured at 490 nm using a microtitre plate reader. The metabolic activity of each drug combination was compared with that of the drug-free biofilms and drug-alone biofilms (control biofilms).

### 2.7. Interpretation of Drug Combination Interaction

Drug combination (200 mg/L of Pcz and 0.5 mg/L of AmB) interaction was classified by the fractional inhibitory concentration index (FICI) [32,33]. The FICI was calculated by the formula: FICI = (A_c_/A_a_) + (B_c_/B_a_), where A_c_ and B_c_ are the metabolic activity of the biofilm cells with antifungal drugs in combination, and A_a_ and B_a_ are the metabolic activity of the biofilm cells with antifungal drugs A and B alone. The interaction was defined as synergistic if the FICI was ≤0.5, additive if FICI was >0.5 and ≤1, indifferent if the FICI was >1 and ≤4, and antagonistic if the FICI was >4.0.

### 2.8. Biofilm Total Biomass Quantification—Crystal Violet Staining

Total biofilm biomass was quantified by Crystal Violet (CV) staining [28]. After biofilm formation, the medium was aspirated, and non-adherent cells removed by washing the biofilms with sterile ultra-pure water. Then, biofilms were fixed with 200 μL methanol, which was removed after 15 min of contact. The microtiter plates were allowed to dry at room temperature, and 200 μL of CV (1% *v*/*v*) was added to each well and incubated for 5 min. The wells were then gently washed twice with sterile, ultra-pure water and 200 μL of acetic acid (33% *v*/*v*) was added to release and dissolve the stain. The absorbance of the obtained solution was read in triplicate in a microtiter plate reader (Bio-Tek Synergy HT, Izasa, Lisbon, Portugal) at 570 nm. The results were presented as absorbance per unit area (Abs/cm^2^). 

### 2.9. Statistical Analysis

Experiments were repeated three times in independent assays. Results were compared using one-way analysis of variance (ANOVA) and Dunnett’s multiple comparisons tests, using GraphPad Prism 7 software. All tests were performed with a confidence level of 95%.

## 3. Results and Discussion

Systemic candidiasis is a growing problem worldwide, especially in critically immunocompromised patients [1,34]. This disease is associated with severe rates of morbidity, mortality, and high economic costs [34,35], hence the choice of the most appropriate therapy is crucial to achieve a clinical cure. 

Therefore, the two drugs studied in this work were selected since both are used in the treatment of candidemia and have different mechanisms of action [2]. 

Table 1 shows the results of the determinations of MIC, MFC, and MBECs for Pcz and AmB for *C. glabrata* ATCC 2001. 

Although the 2017 EUCAST guidelines do not specify an epidemiological cut-off value (ECOFFs) for *C. glabrata* strains regarding Pcz, it is indicated that, for this species, these values are in general higher than for *C. albicans* (sensible ≤ 0.064 mg/L; resistant > 0.064 mg/L). Pfaller et al. [36] and Spregnini et al. [37] suggested similar results: 0.5–1 mg/L and ≤0.03 to 0.5 mg/L, respectively. Thus, the value observed (0.7 mg/L) seems to indicate a tolerant profile [30]. Regarding the AmB MICs, EUCAST points out that strains with values of AmB ≤ 1 mg/L should be considered sensible, which was the case, since the MIC value was 0.25 mg/L (Table 1) [30]. Surprisingly, MIC and MFC determinations for Pcz showed that this drug has a good activity in planktonic cells. Comparing the MIC and the MFC values, they are approximately the same (0.8–0.9 mg/L vs. 0.7 mg/L). In recent works published by our group, the MFC for fluconazole (Flu) and voriconazole (Vcz) for *C. glabrata* ATCC 2001 was almost 200 times and four times higher than the MIC, respectively [28,38]. Thus, the result for Pcz shows an enhanced effectiveness to eliminate planktonic cells, in comparison to Flu and Vcz [28]. Furthermore, the comparable MICs determined for Pcz and Vcz (0.7 and 0.5 mg/L, respectively) confirm the parallel Pcz *in vitro* activity against *Candida* species that have been reported for both drugs [39]. 

**Figure 1:**
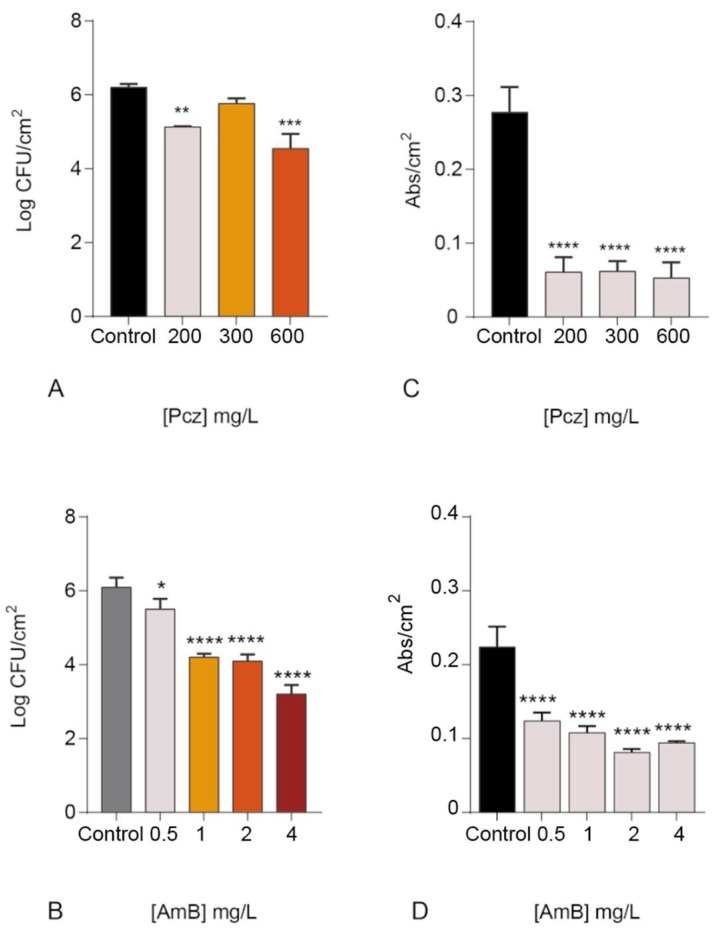
Effect of posaconazole (**A**) and amphotericin B (**B**) on *C. glabrata* ATCC 2001 biofilms. Mean values of the logarithm of colony forming units are normalized by unit of area (Log_10_ CFU/cm^2^). Crystal Violet in a 48-hour-biofilm of *C. glabrata* ATCC 2001 are shown with and without posaconazole (**C**) (200; 300; 600 mg/L) and amphotericin B (**D**) (0.25; 0.5; 1 mg/L). The quantification of the biomass is presented by abs/cm^2^. (* *p* < 0.05; ** *p* < 0.001; *** *p* < 0.0005; **** *p* < 0.0001).

Figure 1 shows the effect of Pcz (Figure 1A,C) and AmB (Figure 1B,D) on *C. glabrata* ATCC 2001 biofilm cells and biomass. Regarding analysis of biofilm cells (enumeration of CFU), Pcz was found to reduce 1 Log_10_ CFU/cm^2^ of the *C. glabrata* ATCC 2001 biofilm cells, when using 200 mg/L (*p* < 0.0005). Curiously, there was no observed improvement in biofilm cells reduction when increasing the dose (Figure 1A). On the other side, AmB displayed a better performance, reducing 2 Log_10_ CFU/cm^2^ from 2 mg/L (Figure 1C). Concerning the biomass decrease, both drugs revealed to be good biofilm reducers. Pcz revealed a statistically significant higher biomass drop with the lower concentration (77.6%, *p* < 0.0001), in comparison with 2 mg/L of AmB (64.2%, *p* < 0.0001) (Figure 1C,D). 

Azoles as Pcz target the ergosterol biosynthetic pathway, specifically the 14-α sterol demethylases, which are encoded by the *ERG11* gene. Thus, these drugs are responsible for blocking the capacity to build and renew sterols in the cellular membranes, changing membrane fluidity and function of vital processes such as signaling, transport, exocytosis, and endocytosis [1,5]. Polyenes as AmB bind to the ergosterol of the fungal cell wall, establishing transmembrane aggregates, pores, and causing membrane depolarization and membrane permeability, which leads to osmotic imbalance and finally cell death [40,41,42]. 

Although not considered a first-line drug for primary candidiasis therapy, Pcz is used in cases of *Candida* infections as a stepping-down therapy for isolates that are susceptible to those agents but not susceptible to Flu (fluconazole-refractory disease) [2]. The concomitant use of azoles and polyenes has been evaluated in some reports in other species [2,43,44,45,46,47,48]. With the purpose of evaluating the antifungal effect (synergism, additive, indifferent, or antagonism) of the combination of Pcz and AmB, clinical concentrations were applied in mature biofilms of *C. glabrata* ATCC 2001. It is important to note that the concentrations used were the MICs of Pcz and AmB, and doses similar to the protocols used in invasive candidiasis [2].

**Figure 2:**
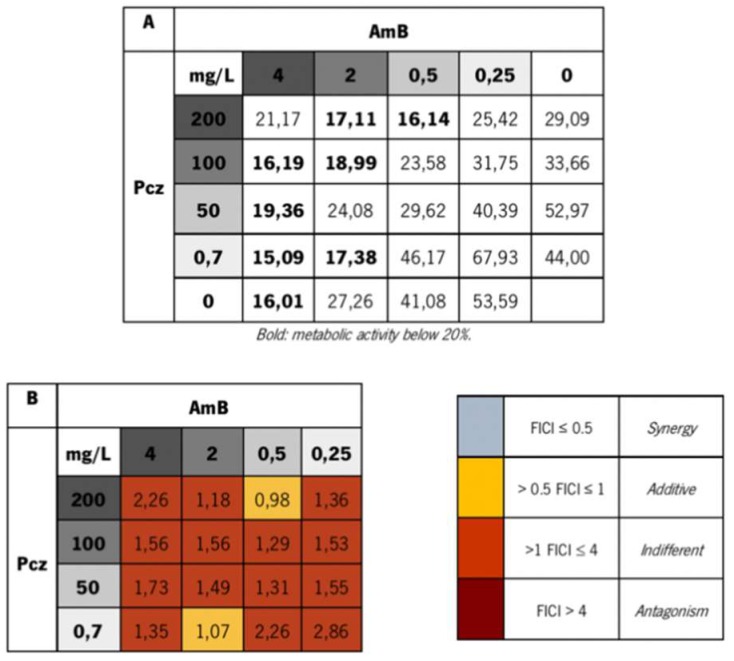
Checkerboard of the combinatory effect of different concentrations of Pcz and AmB. The values are related to the metabolic activity of the biofilms, determined through the XTT assay (**A**) (Bold: metabolic activity below 20%). Calculated FICI range of checkerboard experiments for *C. glabrata* ATCC 2001 (**B**).

Figure 2A displays the checkerboard of the combinatory effect of different concentrations of Pcz and AmB. The values presented are determinations of the metabolic activity of the biofilms, obtained through the XTT assay. These results corroborate CFU counts, since generally, AmB alone showed to have a higher capacity to decrease the metabolic activity of the biofilm cells than Pcz alone (Figure 2A). Further, in terms of metabolic activity, only one combination was able to achieve a 1% improvement than using AmB alone (0.7 mg/L Pcz + 4 mg/L AmB).

**Figure 3:**
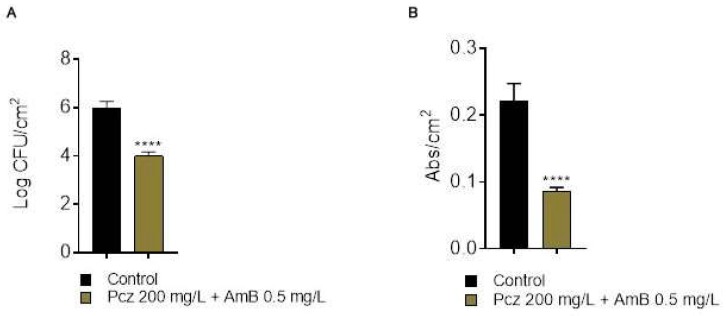
Effect of the combination of posaconazole and amphotericin B (200 + 0.5 mg/L) on *C. glabrata* ATCC 2001 biofilms. Mean values of the logarithm of colony forming units normalized by unit of area (Log_10_ CFU/cm^2^) (**A**). Crystal Violet in a 48-hour-biofilm of *C. glabrata* ATCC 2001, with and without combination of posaconazole and amphotericin B (200 + 0.5 mg/L) (**B**). The quantification of the biomass is presented by abs/cm^2^. (**** *p* < 0.0001).

The determination of the FICI [32,33] values (Figure 2B), showed that the interaction of these drug combinations was, in the majority of the cases, indifferent (>1 FICI ≥ 4), further endorsing the obtained results. Moreover, none of the tested combinations demonstrated a synergistic effect (FICI ≤ 0.5), nor an antagonistic one (FICI > 4), and only two combinations showed a slight additive effect (>0.5 FICI ≤ 1): 0.7 mg/L Pcz + 2 mg/L AmB (FICI = 1.07—Additive/Indifferent) and 200 mg/L Pcz + 0.5 mg/L AmB (FICI = 0.98—Additive). In order to verify the biofilm cell and the biomass eliminations, CFU counts and crystal violet were performed for the combination with the better FICI additive effect (200 mg/L Pcz + 0.5 mg/L AmB) (Figure 3). It was noted that in both combinations, one of the antifungals had to be equal or very near the to the MBEC value to have an additive effect. This fact does not appear to be clinically helpful, since it would also be interesting to reduce the high concentrations needed to eradicate the biofilm, when using two drugs, in order to decrease possible side effects [2].

It was expected that in applying this specific combination, the reduction on biofilm cells were at least 2 Log_10_ CFU/cm^2^ and also that the biofilm biomass would diminish. Nevertheless, none of the objectives were properly achieved. The combination permitted a reduction of 2 Log_10_ CFU/cm^2^ (*p* < 0.0001), which was a better result than the use of Pcz alone, but did not show any clear advantages than the single use of 4 mg/L of AmB, since, in this case, the Log_10_ CFU/cm^2^ reduction was higher (2 in combination vs 2.9 AmB alone in the higher concentration applied). In fact, regarding the results of the CFU counts and comparing both drugs individually, Pcz did not reveal a pronounced effect on the eradication of biofilm cells (i.e., ≥2 Log_10_ CFU/cm^2^), applying the minimum or even the maximum dose (Figure 1A). 

On the other side, the good performance of the AmB alone (Figure 1B) demonstrated that, even though cases of tolerance and resistance have been appearing [14,15,49], AmB is still among the most effective drugs for the treatment of *Candida* infections. Other reports have stated similar results in other *Candida* species [50,51,52]. Considering the biomass reduction, similar results were obtained. The concomitant use of Pcz/AmB led to a good biomass reduction (69.3%), which is an important feature of an antifungal drug in a treatment of an infection caused by biofilms. Yet, a higher reduction was observed when Pcz was used alone (77.6%), but lower than when AmB was used isolated (55.2% for 0.5 mg/L, data not shown) (Figure 1C,D and Figure 2). It is documented that the biofilm structure creates an exceptionally resistant profile to the antifungal drugs [1]. Together, these results validate reports that the biofilm cells are much more resistant than the planktonic and are particularly resistant to azoles (as Pcz), which has also been verified by other authors [16,51,52,53,54,55,56,57,58]. 

In line with our outcomes, Cacciapuoti and colleagues tested several combinations of AmB/Pcz in different *C. albicans* strains. The results revealed cases of synergy but also cases of indifference [43]. Rex and colleagues showed comparable results with AmB and Flu [44]. The authors demonstrated that the combination of AmB/Flu was as effective as higher-dose Flu given alone for patients with candidemia [44]. In opposition, using other drug combinations (AmB, flucytosine, Pcz, caspofungin, or FK506), the authors also reported *in vitro* synergism [2,45]. Additionally, no antagonism was detected in Flu/AmB, saperconazole/AmB, or SCH39304(another azole)/AmB combination in candidiasis studies in murine models [46,47,48,59]. One important feature is that none of these studies were developed with biofilm cells, as the present study has done. 

As the azoles block the ergosterol production which is the target of polyenes, this can reduce the antifungal capacity of the latter (by decreasing the target concentration), which was also described in a number of reports [60,61,62]. Indeed, one of the most important mechanisms of antifungal resistance is the modulated expression of drug targets, as the membrane sterol composition of biofilms’ cells. It is documented that cells from mature biofilms contain a significantly lower concentration of ergosterol, especially during the later phases of biofilm growth, compared to the planktonic cells [63,64,65,66,67,68,69]. Additionally, the azole resistance profile of biofilms of *C. glabrata* and the interference of the antifungals mechanisms of action explained above, may be reasons for the less expressive results in this Pcz/AmB combination, compared with the use of the drugs alone. For this reason, and although there was no clinical isolates evaluation in this report, it is expected that the present results can be extended to them, as these drugs have a different mechanism of action but are both linked to ergosterol and the cellular membrane.

As a final remark, combinational drug therapies have appeared due to the increase of cases of antifungal drug resistance and are usually employed to treat patients with several immunosuppressing diseases (e.g., HIV, cancer). These approaches are an important source of new therapeutic responses and seem to have great potential in fungal infections therapy, but require a careful and constant evaluation of the efficacy of the proposed schemes, before application in clinical practice.

## Figures and Tables

**Table 1:** MICs, MFCs and MBECs determined for posaconazole and amphotericin B for *C. glabrata* ATCC 2001.

Drug	MIC (mg/L)	MFC (mg/L)	MBEC (mg/L)
Posaconazole	0.7	0.8–0.9	>300
Amphotericin B	0.25	1–2	2–4

MIC: minimum inhibitory concentration; MFC: minimum fungicidal concentration; MBEC: minimum biofilm eradication concentration.
